# IDN 5390: an oral taxane candidate for protracted treatment schedules

**DOI:** 10.1038/sj.bjc.6600784

**Published:** 2003-03-18

**Authors:** G Pratesi, D Laccabue, C Lanzi, G Cassinelli, R Supino, M Zucchetti, R Frapolli, M D'Incalci, E Bombardelli, P Morazzoni, A Riva, F Zunino

**Affiliations:** 1 Istituto Nazionale Tumori, via Venezian 1, 20133 Milano, Italy; 2 Istituto Mario Negri, via Eritrea 62, 20157 Milano, Italy; 3 Indena S.p.A., Viale Ortles 12, 20139 Milano, Italy

**Keywords:** taxanes, IDN 5390, angiogenesis inhibitors, oral route

## Abstract

The recognition of the antiangiogenic properties of taxanes provides a basis for novel therapeutic approaches. A prolonged exposure to low drug concentrations has been proposed to be the most suitable approach to exploit the antiangiogenic potential of cytotoxic agents. Such schedule is required to target preferentially slowly dividing endothelial cells. The protracted use of taxanes could benefit from the availability of a taxane endowed with a favourable tolerability profile. Among compounds of a novel series of C-seco taxanes, IDN 5390 was originally selected on the basis of its potent antimotility activity and poor cytotoxicity on endothelial cells. The aim of the study was to investigate the preclinical pharmacologic profile of IDN 5390 in a variety of human tumour xenografts, including ovarian and colon carcinoma and a glioblastoma. IDN 5390, delivered by s.c. injection, daily for 5 days per week, exhibited a high activity against all tumours investigated (tumour growth inhibition was always >85%) in the range of well-tolerated doses. The maximum tolerated dose/injection (MTD), with no signs of systemic or local vesicant toxicity, was 120 mg kg^−1^. In contrast, paclitaxel, delivered according to the same schedule, exhibited a variable antitumour efficacy associated with a substantial local toxicity (MTD=10 mg kg^−1^). Considering the remarkable efficacy of IDN 5390 delivered s.c. by protracted treatment schedule, the oral route of administration was further investigated, as the most suitable for daily treatment. Indeed, a good bioavailability of oral IDN 5390 was found. Oral IDN 5390 maintained a substantial efficacy against human tumour xenografts, including paclitaxel-resistant tumours, without loss of potency with respect to s.c. administration. In conclusion, the therapeutic advantages of IDN 5390, over paclitaxel, in protracted daily treatment schedules are represented by the oral efficacy and the high tolerability, which are favourable features to exploit the antiangiogenic potential and to design combinations with other effective agents.

In the field of antineoplastic chemotherapy, taxanes (paclitaxel and docetaxel) represent an important class of compounds, with broad activity in solid tumours (Rowinsky, 1997). Taxanes are tubulin-binding agents, characterised by a unique mechanism of action, that is, microtubule stabilisation (Horwitz *et al*, 1993), in contrast to microtubule depolymerisation promoted by vinca alkaloids and colchicine (Jordan *et al*, 1991).

Microtubule-destabilising agents have been recently investigated as antivascular compounds (Iyer *et al*, 1998; Blakey *et al*, 2002), and an antiangiogenic potential has been reported for paclitaxel (PTX) and docetaxel (Belotti *et al*, 1996; Klauber *et al*, 1997; Lau *et al*, 1999; Schimming *et al*, 1999; Sweeney *et al*, 2001; Cassinelli *et al*, 2002). The antiangiogenic activity of PTX has been ascribed mainly to its ability to inhibit endothelial cell motility, rather than proliferation (Belotti *et al*, 1996) and to downregulate angiogenesis-related growth factors (Cassinelli *et al*, 2002). In order to target preferentially endothelial cells in *in vivo* models, a dosing schedule of the drug by short intervals and no interruption has been indicated as effective, in contrast to conventional schedules (i.e. maximum tolerated doses with extended resting periods) (Hanahan *et al*, 2000). By delivering conventional agents according to such ‘an antiangiogenic schedule’, a strong antitumour efficacy was achieved, even against chemoresistant tumours (Browder *et al*, 2000; Klement *et al*, 2000). The protracted use of cytotoxic agents may be hampered by a relevant toxicity. To identify novel molecules endowed with a favourable profile of tolerability in protracted treatment schedules, a series of taxane analogues was synthesised (Appendino *et al*, 1997; Distefano *et al*, 1998), and the C-seco taxane IDN 5390 was selected for preclinical development (Pratesi *et al*, 2002; Taraboletti *et al*, 2002).

The aim of the study was to further characterise the biological properties of IDN 5390 and to investigate its preclinical pharmacological profile in a series of human tumour xenografts, including PTX-resistant tumours. The oral or s.c. daily administration of IDN 5390 was highly effective against all the tumour models investigated.

## MATERIALS AND METHODS

### Drugs

**Figure 1:**
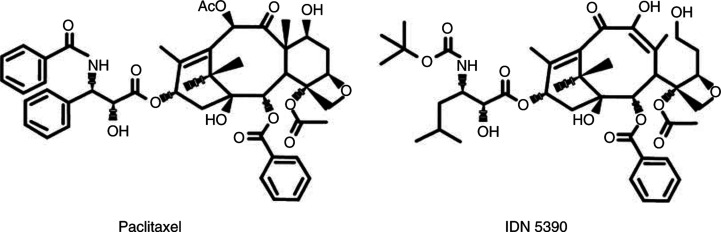
Chemical structure of paclitaxel and IDN 5390.

IDN 5390 (13-(*N*-Boc-*β*-isobutylisoserinyl)-10*β*-dehydro-*C*-seco-10-deacetylbaccatin III) and PTX (Figure 1

), used as reference drug, were provided by Indena S.p.a. (Milan, Italy).

### *In vitro* studies

Drugs were dissolved in DMSO and freshly diluted in medium before use. The antiproliferative activity was evaluated on A2780 (human ovarian carcinoma) and its derived drug-resistant cell lines (A2780/DX, A2780/DDP), IGROV-1/Pt1 (human ovarian carcinoma), GBM (human glioblastoma) and MX-1 (human mammary carcinoma). All cell lines, except MX-1, were maintained in RPMI 1640 (Bio-Whittaker, Verviers, Belgium) supplemented with 10% fetal calf serum (Life Technologies, Gaithersburg, MD, USA). MX-1 cells were maintained in McCoy's medium (Life Technologies, Gaithersburg, MD, USA) supplemented with 10% fetal calf serum. For experiments, all cell lines were seeded into six-well plates in duplicate and treated, 24 or 72 h (only MX-1 cells) after seeding, with solvent or different concentrations of taxanes. Adherent cells were counted 72 h after the beginning of treatment, by a cell counter (Coulter Electronics, Luton, UK) or detected by the Sulforhodamine B colorimetric assay (only GBM cells). The IC_50_ was defined as the drug concentration causing a 50% decrease in cell number over that of untreated controls.

For cell cycle analysis, after 24 h of treatment, GBM adherent cells were trypsinised and fixed in 70% ethanol. Cell cycle perturbations were measured on propidium iodide-stained cells by flow cytometry (Supino *et al*, 2001).

For tubulin polymerisation assay, IGROV-1/Pt1 cells were treated with PTX or IDN 5390 at the indicated concentrations for 24 h. Cells were then processed as previously described (Cassinelli *et al*, 2001) in order to separate cellular fractions containing soluble cytosolic or polymerised cytoskeletal tubulin. Samples were electrophoresed by sodium dodecyl sulphate (SDS)–PAGE (10% resolving gel and 3% stacking gel) and blotted onto nitrocellulose membranes. The distribution of tubulin was analysed by immunoblotting, using a rabbit polyclonal antitubulin antibody (BioMakor, Revohot, Israel).

To investigate taxane-induced modulation of Raf-1 and Bcl-2, IGROV-1/Pt1 cells were exposed to the indicated concentrations of drug for 24 h. Expression of vascular endothelial growth factor (VEGF) and basic fibroblast growth factor (bFGF) was detected in GMB cells after 72 h of drug exposure. Floating and adherent cells were collected and whole-cell extracts were prepared by lysing cells in SDS sample buffer (62.5 mM Tris-HCl, pH 6.8, 2% SDS, 10% glycerol, 5% *β*-mercaptoethanol, 0.001% bromophenol blue) with 1 mM PMSF, 10 *μ*g ml^−1^ pepstatin, 100 kIU aprotinin, 12.5 *μ*g ml^−1^ leupeptin, 1 mM sodium orthovanadate and 1 mM sodium molybdate. Equal amounts of proteins were fractionated by SDS–PAGE and blotted on nitrocellulose sheets. Filters were incubated with mouse monoclonal anti-Bcl-2 antibody or with the rabbit polyclonal antibodies anti-Raf-1, anti-VEGF or anti-bFGF (Santa Cruz Biotechnology, CA, USA). Immunoreactive bands were visualised by the Pierce Super Signal system (Pierce, Rockford, IL, USA).

### *In vivo* studies

All the experiments were carried out using female athymic nude CD-1 mice, 8–11 weeks old (Charles River, Calco, Italy). The mice were maintained in laminar flow rooms with constant temperature and humidity. Experimental protocols were approved by the Ethics Committee for Animal Experimentation of the Istituto Nazionale per lo Studio e la Cura dei Tumori (Milan, Italy) according to the United Kingdom Coordinating Committee on Cancer Research Guidelines (Workman *et al*, 1998).

Taxanes were dissolved by adding absolute ethanol and Cremophor ELP (both 5% of the final volume), in that sequence, on a magnetic stirrer. Details of the procedure have been already reported (Polizzi *et al*, 1999). After dilution, the drug solutions were always kept on ice. For the oral delivery of IDN 5390 a different solvent was used, that is, Polysorbate 80 in 0.9% NaCl, because it allowed a more favourable relative drug bioavailability than Cremophor ELP in the mouse (data not shown), and is more appropriate for clinical development. A stock solution of IDN 5390 in Polysorbate 80 was provided by Indena S.p.a. (Milano, Italy) and, just before use, it was diluted, by slowly adding 0.9% NaCl and occasionally stirring the solution (final Polysorbate 80: 10%, v v^−1^). With such procedures, drug solutions of 3.6 and 9 mg ml^−1^ were prepared for PTX and IDN 5390, respectively, corresponding to 36 and 90 mg kg^−1^, for an administration volume of 10 ml kg^−1^ of body weight (BW). When higher doses were to be given, larger volumes were injected.

The human tumour lines investigated in the study were selected for their variable degrees of sensitivity to PTX, that is, highly sensitive, MX-1 mammary and A2780/DDP ovarian carcinomas, and resistant, IGROV-1 (natural resistance) and INT.ACP/PTX (acquired resistance) ovarian carcinomas. The INT.ACP/PTX tumour line was derived from a single A2780/DDP tumour that did not respond to PTX treatment. Tumour lines were maintained *in vivo* by successive s.c. transplants of tumour fragments in animal flanks.

For chemotherapy experiments, tumour fragments (about 2 × 2 × 2 mm), obtained from tumour lines, were implanted s.c. Each control or drug-treated group included five or six mice bearing bilateral s.c. tumours. Tumours were implanted on day 0, and tumour growth was followed by biweekly measurements of tumour diameters with a vernier caliper. Tumour weight (TW) was calculated according to the formula: TW (mg)=tumour volume (mm^3^)=d^2^ × D/2, where *d* and *D* are the shortest and the longest diameter, respectively. Different treatment routes (i.v., s.c. or p.o.) and schedules (daily or intermittent) were investigated. Treatment started at different times after tumour implant (see Results). Control mice were injected with the solvent solution.

The efficacy of the drugs was assessed as follows: (a) TWI% in drug-treated *vs* control mice expressed as: TWI%=100 − (mean TW treated/mean TW control × 100), evaluated during or after drug treatment; (b) Log_10_ cell kill (LCK) calculated by the formula: LCK=(*T−C*)/3.32 × DT, where *T* and *C* are the mean times (in days) required for treated and control tumours, respectively, to reach a predetermined weight and DT is tumour doubling time; an LCK value greater than 1 is indicative of an active compound; and (c) complete response (CR), that is, no evidence of tumour at the end of experiments. Experimental groups were eliminated when mean TW was about 1.5±0.5 g, or after 100 days.

For statistical analysis, tumour weights in different groups of treated mice were compared on day of TWI% evaluation, by Student's *t*-test (two-tailed). *P*⩽0.05 was considered significant.

The tolerability of the compound was assessed in tumour-bearing mice as follows: (a) lethal toxicity, that is, any death in treated mice without or with small (⩽300 mg) tumours, or occurring before any control death; (b) body weight loss % (BWL%)=100–(BW on day *x*/BW on day 1 × 100), where *x* represents a day after or during the treatment period. The maximal BWL values are reported; and (c) vesicant toxicity defined as the appearance of ulcers and/or inflammation at the injection site where the drugs were delivered s.c.

### Pharmacokinetic study

In order to assess the bioavailability of IDN 5390, a pharmaco-kinetic study was performed in female CDF1 mice (Charles River Italia, Calco, Italy). IDN 5390, formulated in Polysorbate 80 and diluted in 0.9% NaCl solution, immediately before treatment, was administered p.o. or i.v. at the dose of 120 mg kg^−1^. Blood samples were taken (four mice per group) from the retro-orbital plexus, under diethylether anaesthesia at 5, 15 and 30 min and at 1, 2 and 4 h.

IDN 5390 levels were determined in plasma according to the high-performance liquid chromatography (HPLC) method of Zaffaroni *et al* (2002). Briefly, plasma samples (0.4 ml) were spiked with 1 *μ*g IDN 5517, as internal standard, and with 0.7 ml of 0.2 M ammonium acetate buffer, pH 4.5, and extracted by solid-phase extraction on a cyano cartridge (Waters, Millford, MA, USA). HPLC analyses were carried out on a Symmetry C18 column (3.5 *μ*m, 4.6 × 150 mm; Waters, London, UK), with UV detection of the analytes at 227 nm. The lower limit of quantitation was of 50 ng ml^−1^.

Pharmacokinetic parameters were calculated by using a nonlinear fitting program (Sacchi-Landriani *et al*, 1983). The experimental areas under the concentration–time curve (AUC_exp_) of IDN 5390 were calculated by the trapezoidal rule. Extrapolations to infinity (AUC_inf_) were obtained by dividing the concentration at the last experimental points by the elimination constant (*K*_e_). The terminal half-lives (*T*_1/2_) were calculated using the formula *T*_1/2_=0.693/*K*_e_. Clearance (Cl_p_) and volume of distribution (*V*_d_) were obtained from the following relation: Cl_p_=Dose/AUC_ext_ and *V*_d_=Cl_p_/*K*_e_, respectively. The bioavailability (*F*) was derived from the ratio: *F*=AUC_p.o._/AUC_i.v._ 100.

## RESULTS

### Cellular pharmacology

In a panel of human tumour cell lines, IDN 5390 exhibited a modest reduction of cytotoxicity (2–3-fold) as compared to PTX (Table 1

**Table 1:** Antiproliferative activity of taxanes

		**IC50 (ng/ml)^a^**			
**Cell line**	**Tumour type**	**IDN 5390**	**PTX**		
A2780	Ovarian carcinoma	23±0.4	8.1±0.9
A2780/DX	Ovarian carcinoma	334	313
A2780/DDP	Ovarian carcinoma	31.8±5.5	11.8±1.4
IGROV-1/Pt	Ovarian carcinoma	61.5±4.5	36.1±5.5
MX-1	Mammary carcinoma	34.5±7.8	12.7±2.7
GBM	Glioblastoma	12.9±2.4	3.2±1.2

a Cells were treated for 72 h. The antiproliferative effect was evaluated by the Sulforhodamine B colorimetric assay for the GBM cell line or by cell counting for the other cell lines. IC_50_: drug concentration inhibiting 50% of cell proliferation (mean±s.d., from three different experiments).

). In A2780/DX cells (which express a typical MDR phenotype mediated by P-gp), both agents exhibited very low cytotoxicity, thus suggesting that both agents are substrates for P-gp.

**Figure 2:**
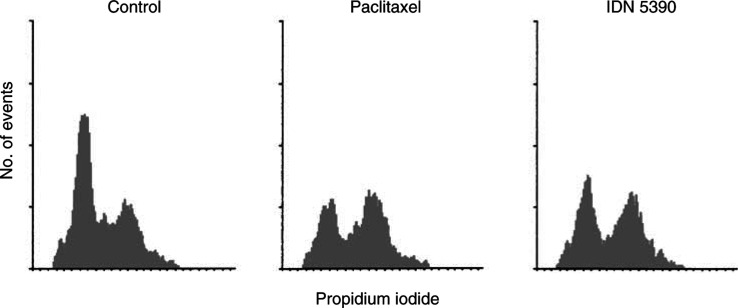
Cell cycle distribution in GBM cells treated with taxanes at the IC_50_. After 24 h of treatment, cells were harvested, fixed, stained with propidium iodide and analysed by flow cytometry. One experiment representative of two is reported.

Cell cycle distribution analysis of GBM cells treated with equitoxic concentrations (IC_50_) of the two taxanes, showed a similar increase of cells in G2/M phase 24 h after treatment (Figure 2

).

**Figure 3:**
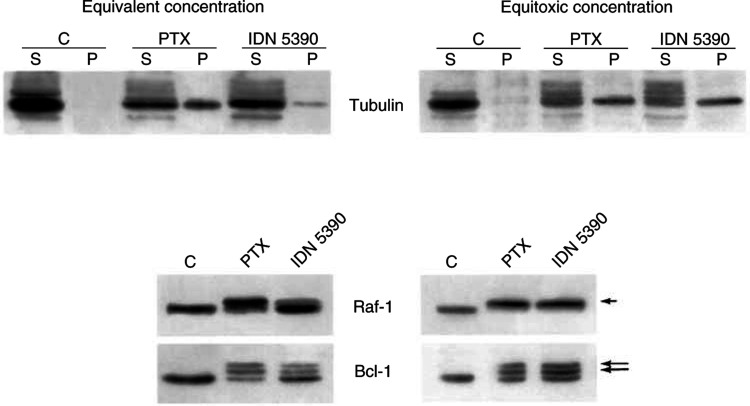
Effects of taxanes on tubulin polymerisation and on Raf-1/Bcl-2 phosphorylation in IGROV-1/Pt1 cells. Cells were treated for 24 h at equivalent (100 ng ml^−1^) or equitoxic (IC_80_) concentrations (PTX, 100 ng ml^−1^; IDN 5390, 300 ng ml^−1^). Cellular fractions containing soluble cytosolic (S) or polymerised cytoskeletal (P) tubulin were subjected to immunoblotting with antitubulin antibodies. Whole-cell extracts were processed for immunoblotting with anti-Raf-1 or anti-Bcl-2 antibodies. The phosphorylated forms of Raf-1 and Bcl-2 are indicated by arrows.

Based on the observed interference on cell cycle progression, comparison among IDN 5390 and PTX for the ability to induce tubulin polymerisation and phosphorylation of Bcl-2 and Raf-1 was performed, after 24 h exposure. According to the lower antiproliferative potency, IDN 5390 was less effective than the parent drug in promoting tubulin polymerisation at the same concentration (100 ng ml^−1^) in IGROV-1/Pt cells. However, a comparable extent of tubulin polymerisation was found in cells treated with equitoxic concentrations (IC_80_) of IDN 5390 or PTX (300 and 100 ng ml^−1^, respectively). Again, the analysis of Bcl-2 and Raf-1 phosphorylation, events associated with taxane-induced mitotic arrest (Ling *et al*, 1998), indicated comparable levels of phosphorylation induced by equitoxic concentrations of the two drugs (Figure 3

).

**Figure 4:**
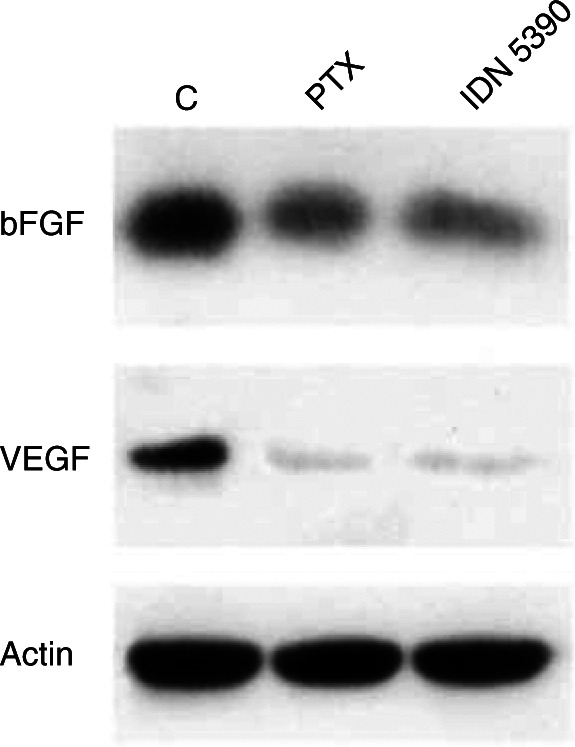
Effects of taxanes on VEGF and bFbF expression in GBM cells. Cells were treated for 72 h at equitoxic (IC_80_) concentrations (PTX, 17 ng ml^−1^; IDN 5390, 40 ng ml^−1^). Whole-cell extracts were processed for immunoblotting with anti-VEGF or anti-bFGF antibodies.

The effects of the C-seco derivative on the expression of the two main angiogenic factors, VEGF and bFGF, were examined in GBM cells. After 72 h of exposure to 40 ng ml^−1^ IDN 5390 (corresponding to its IC_80_), both angiogenic factors were downregulated, the effect on VEGF being more pronounced (Figure 4

). The equitoxic concentration of PTX (17 ng ml^−1^) showed similar effects.

### *In vivo* studies

The optimal PTX regimen for i.v. treatment of human tumours xenografted in mice is the intermittent administration every fourth day (q4d) for four times, with the MTD 54 mg kg^−1^ inj^−1^ (inj = injection) (Polizzi *et al*, 1999). Based on the relative cytotoxic potency observed in cell systems, doses 2–3-fold higher were delivered for i.v. IDN 5390 (i.e. 90 and 120 mg kg^−1^), q4d × 4 times, to mice bearing the A2780/DDP human tumour xenograft (Table 2

**Table 2:** Antitumour activity of taxanes on A2780/DDP ovarian tumour xenograft

	**Treatment**						
**Drug**	**Route**	**Schedule^a^**	**Dose (mg kg^−1^)**	**Max TWI%^b^ (days)^c^**	**LCK^d^ (1 g)**	**Max BWL^e^ (%)**	**Tox/Tot^f^**
IDN 5390	i.v.	q4d × 4	90	17 (18)	0.2	0	0/5
	i.v.	q4d × 4	120	46 (18)	0.3	3	0/5
PTX	i.v.	q4d × 4	54	94 (18)	2.1	5	0/5
							
IDN 5390	s.c.	qd × 5/w × 2w	120	92 (27)	1.8	2.5	0/5
PTX	s.c	qd × 5/w × 2w	10	62 (27)	0.2	0	0/5

a Drugs were delivered every fourth day for four doses (q4d × 4) or daily (qd) for five doses each week for 2 weeks, starting when mean tumour weight was about 100 mg.

b Optimal tumour weight inhibition (%) in treated *vs* control mice. Values in parentheses represent the day of observation.

c Days are always calculated from the day of tumour inoculum (day 0).

d Log_10_ cell kill in treated tumours, calculated to a mean TW of 1 g.

e Maximum body weight loss (%).

f Number of toxic death/total number of mice in the group.

). In contrast to PTX, the analogue resulted quite inactive. Thus, an alternative schedule with a more frequent treatment (daily for 5 days per week, qd × 5/w) was investigated. The s.c. route was preferred to the i.v., as it was more suitable for protracted daily treatments in mice. According to such schedule, after 2 weeks of treatment (weekend excluded), IDN 5390 (120 mg kg^−1^ inj^−1^) was as active as PTX at its best regimen. In contrast, daily PTX was much less effective at the dose of 10 mg kg^−1^ inj^−1^, which was the maximal tolerated as a consequence of the local vesicant toxicity. Indeed, higher PTX doses or more protracted treatment resulted in severe ulcerations in most mice. This side effect was not observed in IDN 5390-treated mice.

Based on these findings, the daily schedule was chosen for further experiments with IDN 5390. Against the MX-1 mammary carcinoma, which is highly responsive to PTX, various dose levels of IDN 5390 were investigated by the s.c. route, with the treatment beginning the day after tumour inoculum (Table 3

**Table 3:** Antitumour activity of taxanes on MX-1 mammary tumour xenograft

	**Treatment**						
**Drug**	**Route**	**Schedule^a^**	**Dose (mg kg^−1^)**	**Max TWI%^b^ (days)**	**LCK^d^ (1 g)**	**Max BWL^e^ (%)**	**Tox/Tot**
IDN 5390	s.c.	qd × 4–5/w × 3w	60	83(29)	1.4	3	0/3
	s.c.	qd × 4–5/w × 3w	90	97(29)	2.7	3.5	0/5
	s.c.	qd × 4–5/w × 3w	120	93–96*(25)	1.7–3.1	6–7.5	1/10
	s.c.	qd × 4	150	N.D.	N.D.	N.D.	3/5
	p.o.	qd × 4–5/w × 3w	120	82(25)	1.1	5	0/5
PTX	i.v.	q4d × 4	54	100(45)	>4.3	8	0/4

a Drugs were delivered every fourth day for four doses (q4d × 4) or daily for four or five doses each week for 1–3 weeks, from day 1. See footnotes b–f of Table 2.

* *P*<0.05 *vs* p.o.-treated tumours, by Student's *t*-test.

). After 3 weeks of treatment (weekend excluded), all doses were highly effective in inhibiting tumour growth (TWI always >80%) and comparable effects were achieved with 90 and 120 mg kg^−1^. In spite of the prolonged treatment (3 weeks), the dose of 120 mg kg^−1^ inj^−1^ was well tolerated and devoid of local vesicant toxicity. The dose level of 150 mg kg^−1^ resulted highly toxic (three out of five lethal toxicity) after only four administrations. Considering the impressive efficacy of the protracted treatment schedules with IDN 5390 s.c., the oral route of administration was investigated, as the most suitable for daily treatment. The dose level of 120 mg kg^−1^ was highly effective in inhibiting tumour growth even with oral administration.

**Figure 5:**
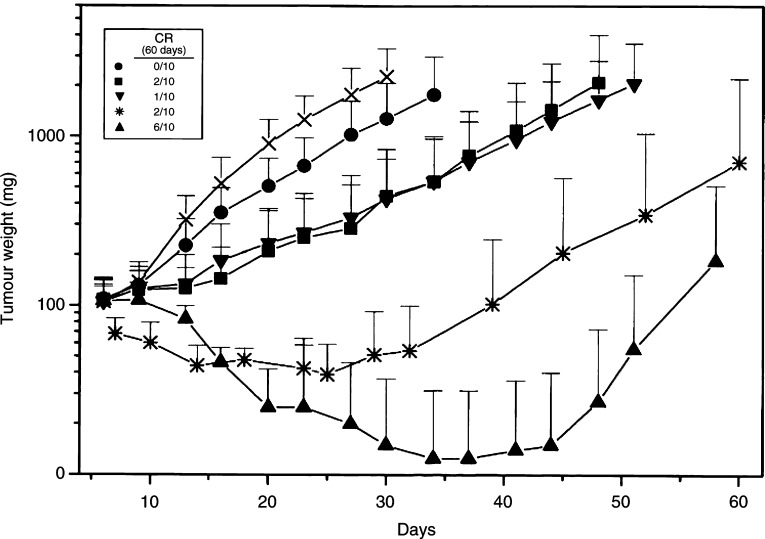
Growth curves of MX-1 human mammary carcinoma xenografted in nude mice after treatment with IDN 5390, qd × 5d/w: (•) 2 × 30 mg kg^−1^ (qd × 3w), p.o.; (▪) 2 × 60 mg kg^−1^ (qd × 3w), p.o., (▴) 2 × 90 mg kg^−1^ (qd × 5w), p.o.; (▾) 120 mg kg^−1^ (qd × 3w), p.o.; (*) 120 mg kg^−1^ (qd × 3w), s.c.; (×) untreated control. Inset: number of complete response/total number of tumours.

The complete lack of any toxic effect suggested the possibility to further increase the frequency with oral treatment. MX-1 tumour-bearing mice were treated p.o. with various (i.e. 30, 60 and 90 mg kg^−1^inj^−1^) dose levels of IDN 5390, twice a day (2 ×), starting when tumours averaged 100 mg (Figure 5

). All doses resulted well tolerated. The low dose, 30 mg kg^−1^, was not very effective. The daily dose of 120 mg kg^−1^ for 3 weeks was very effective when delivered either by a single or by two fractionated treatments (2 × 60 mg kg^−1^), and TWI and LCK values of the two groups were not significantly different. The most relevant result of the study was the impressive efficacy achieved by the treatment with the dose of 2 × 90 mg kg^−1^. The dose was completely tolerated (11% max BWL, no lethal toxicity) for up to 5 weeks for a total of 50 treatments, that is a cumulative dose of 4500 mg kg^−1^. The activity was extremely high and six out of 10 tumours exhibited CR at the end of the experiment (100 days).

The efficacy of the daily IDN 5390 was tested against two PTX- resistant ovarian human tumour xenografts, that is, the IGROV-1 (natural resistance, likely related to P-gp expression) and the INT-ACP/PTX (acquired resistance to both cisplatin and PTX). The oral dose of 120 mg kg^−1^, daily for 3 weeks (weekend excluded), was well tolerated and active against the IGROV-1 tumour, whereas PTX at its best regimen was inactive, achieving a TWI of 30% (Table 4

**Table 4:** Antitumour activity of taxanes on PTX-resistant ovarian tumour xenografts

	**Treatment**						
**Drug**	**Route**	**Schedule^a^**	**Dose (mg kg^−1^)**	**TWI%^b^ (day 27)**	**LCK^d^ (1 g)**	**Max BWL^e^ (%)**	**Tox/Tot**
*IGROV-1*							
IDN 5390	p.o.	qd × 5/w × 3w	120	73	2.2	13	0/4
PTX	i.v.	q4d × 4	54	30	0.2	17	1/5
*INT.ACP/PTX*							
IDN 5390	p.o.	qd × 5/w × 3w	2 × 90	66	0.8	9	0/6
	p.o.	qd × 5/w × 2w	2 × 120	87	1.4	15	1/5
	p.o.	qd × 5/w × 2w	180	96*	1.9	10	2/5
PTX	s.c.	qd × 5/w × 2w	1.25	12	0	0	0/4
	s.c.	qd × 5/w × 2w	2.5	26	0	0	0/5
	s.c.	qd × 5/w × 2w	5	−10	−0.2	0	0/5
	s.c.	qd × 5/w × 2w	10	43	0.1	2	0/5
	i.v.	q4d × 3	54	46	0.3	14.5	1/5

a Drugs were delivered: every fourth day for 3/4 doses (q4d × 3/4); once daily (qd) or twice (2 ×) for four or five doses each week, for 2 or 3 weeks, starting when mean tumour weight was about 75 – 100 mg. See footnotes b–f of Table 2.

* *P*<0.05 (by Student's *t*-test) *vs* 2 ×90 mg kg^−1^ day^−1^ treated mice.

). Against the INT-ACP/PTX human tumour xenograft, the dose of 2 × 90 mg kg^−1^ (twice a day) showed only a partial efficacy, whereas a strong efficacy (96% TWI) was achieved by a daily injection of 180 mg kg^−1^. However, such dose was toxic after 2 weeks of treatment. Although highly active (87% TWI), the treatment with two injections per day of 120 mg kg^−1^ for 2 weeks was not tolerated (one toxic death and 15% BWL). Against the INT-ACP/PTX tumour, PTX was inactive (TWI <50%) when administered either i.v. (best regimen) or s.c. (daily treatment) (Table 4).

### Pharmacokinetic study

Figure 6

**Figure 6:**
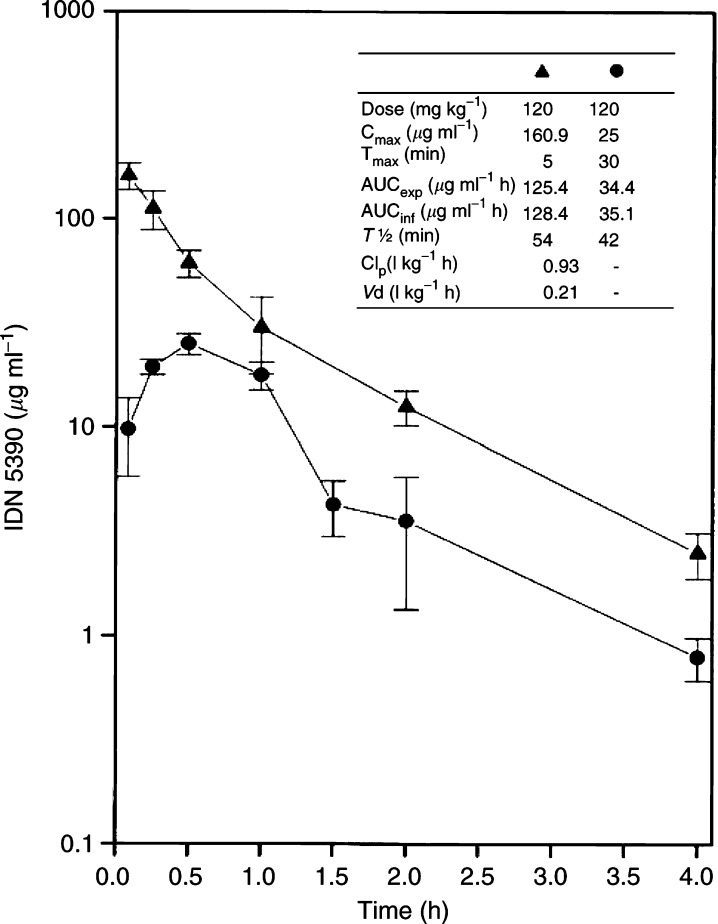
Pharmacokinetics profile of IDN 5390 in CDF1 mice. Comparison of drug plasma levels after treatment with 120 mg kg^−1^ i.v. (▴) or p.o. (•). Means±s.d. are reported from four mice per group. Inset: main parameters.

shows the plasma levels of IDN 5390 determined in CDF1 mice after p.o. or i.v. treatment with 120 mg kg^−1^ of the drug, and summarises the main derived pharmacokinetic parameters. Following i.v. administration, the drug was rapidly distributed and eliminated with a terminal half-life of 54 min. After oral treatment, IDN 5390 was rapidly absorbed, achieving a peak plasma concentration of 25.0 *μ*g ml^−1^ at 30 min. As observed after the i.v. administration, the drug was rapidly eliminated with a terminal half-life of 42 min. A comparison of the AUCs obtained after oral and i.v. administration indicated that IDN 5390 possessed a bioavailability of 27.3%.

## DISCUSSION

The results presented in the paper clearly indicated that the novel C-seco derivative IDN 5390, was well tolerated in protracted multiple dose regimens. The excellent tolerability of IDN 5390 was an unexpected finding, since in *in vitro* systems the derivative is only slightly less cytotoxic than PTX and the effects on tubulin polymerisation are comparable to those of PTX at equitoxic concentrations. Again, the effects of IDN 5390 and PTX on pathways involving Bcl-2 and Raf phosphorylation were comparable, thus suggesting a common mechanism of action at the cellular level, with tubulin as the primary cellular target and activation of biochemical pathways that typically characterise the cellular response to microtubule damage (Cassinelli *et al*, 2001; Lanzi *et al*, 2001).

The most important finding of the present study was the peculiar pharmacological behaviour of IDN 5390 in *in vivo* systems, Indeed, IDN 5390 delivered according to an intermittent schedule, considered as an optimal regimen for PTX in preclinical models (Polizzi *et al*, 1999), was inactive against a responsive human ovarian carcinoma xenograft, the A2780/DDP, even at doses higher than those of PTX. However, the growth of the same tumour was strongly inhibited by IDN 5390 when the compound was delivered more frequently. The daily administration of IDN 5390 was consistently highly effective against all the tumour xenografts investigated, including PTX-resistant tumours. Interestingly, the analogue was effective (TWI>70%, LCK: 2.2) even against the IGROV-1 ovarian tumour xenograft, which is naturally resistant to PTX possibly because of Pgp 170 expression (De Cesare *et al*, 2001). Since in cell systems, IDN 5390 was unable to overcome Pgp-mediated resistance (Table 1), mechanisms other than a direct cytotoxicity against tumour cells seem to contribute to its antitumour efficacy *in vivo*.

The unique profile of tolerability and antitumour activity of IDN 5390 is likely related to still unrecognised changes in drug–cellular interactions that may be critical in modulating the toxic and therapeutic effects of taxanes. The opening of the ring at C-7 and C-8 is consistent with the reduction of cytotoxic potency; however, such modulation does not account for the tolerability of substantially higher dose levels than with PTX. When delivered by daily s.c. administration, the MTD for a prolonged period (at least 3 weeks) was 120 mg kg^−1^ per treatment. The same dose level was tolerated and active even when delivered p.o., thus suggesting an adequate bioavailability of the compound. At the dose level investigated in the study, about 30% of IDN 5390 was absorbed after oral administration. Indeed, the pharmacokinetic studies indicated a rapid absorption, distribution and elimination (*T*_1/2_<1 h) of IDN 5390 in mice. Accordingly, when two treatments per day (2 × 90 mg kg^−1^) were delivered p.o., again no toxic effects were observed in treated mice up to at least 5 weeks (50 treatments), indicating the absence of drug accumulation. The good efficacy of the oral treatment represents a pharmacological advantage of IDN 5390 over PTX, which is poorly absorbed by such administration route (Sparreboom *et al*, 1997).

In addition to the lack of systemic toxicity, no symptoms of neurotoxicity were evident in IDN 5390-treated mice. Moreover, no local vesicant toxicity was observed with the s.c. delivery of IDN 5390 at any dose. In contrast, local toxicity was frequent and persistent with PTX s.c., and the maximum dose level tolerated was 10 mg kg^−1^ day^−1^ for 2 weeks. In contrast to PTX, which exhibited a variable activity against the examined tumour models with the daily treatment, the efficacy of IDN 5390 was consistently high in all tumours. Such a pharmacological profile suggests a mechanism of antitumour activity in part different from that of PTX. A plausible explanation for the unusual behaviour of IDN 5390 could be a contribution of antiangiogenic properties of the C-seco derivative to the therapeutic efficacy. The ability of IDN 5390 to downregulate the expression of angiogenic factors has been documented in our study (Figure 4). Very high dose levels could be safely delivered for repeated weeks, possibly allowing a strong and prolonged inhibitory effect of angiogenic growth factors. It seems likely that such property, in addition to the unique ability to inhibit endothelial cell motility (Taraboletti *et al*, 2002), might contribute to the antitumour effect of IDN 5390 *in vivo*. Moreover, prolonged chronic treatment schedules have been reported as the most suitable for exploiting the antiangiogenic effect of cytotoxic drugs in *in vivo* systems (Browder *et al*, 2000; Hanahan *et al*, 2000; Klement *et al*, 2000). Preclinical and clinical experiences with PTX support the hypothesis (Miller *et al*, 2001) and such schedule resulted as the best one for the antitumour efficacy of IDN 5390. A relation between angiogenesis and tumour growth is complex in *in vivo* systems (Hahnfeldt *et al*, 1999) and will be addressed in a further study.

In conclusion, the novel taxane analogue IDN 5390 is highly effective in a panel of human tumour xenografts, including PTX-resistant tumours. The discrepancy between its *in vitro* and *in vivo* potency, as well as the requirement for frequent and protracted administration for drug efficacy seem to indicate a host-mediated contribution, more than a direct cytotoxic effect, in its antitumour efficacy. Moreover, the unexpected tolerability and the good bioavailability indicate a peculiar pharmacological behaviour and the interest for further development of the compound administered orally.
